# Rare Variants in 48 Genes Account for 42% of Cases of Epilepsy With or Without Neurodevelopmental Delay in 246 Pediatric Patients

**DOI:** 10.3389/fnins.2019.01135

**Published:** 2019-11-08

**Authors:** Ana Fernández-Marmiesse, Iria Roca, Felícitas Díaz-Flores, Verónica Cantarín, Mª Socorro Pérez-Poyato, Ana Fontalba, Francisco Laranjeira, Sofia Quintans, Oana Moldovan, Blanca Felgueroso, Montserrat Rodríguez-Pedreira, Rogelio Simón, Ana Camacho, Pilar Quijada, Salvador Ibanez-Mico, Mª Rosario Domingno, Carmen Benito, Rocío Calvo, Antonia Pérez-Cejas, Mª Llanos Carrasco, Feliciano Ramos, Mª Luz Couce, Mª Luz Ruiz-Falcó, Luis Gutierrez-Solana, Margarita Martínez-Atienza

**Affiliations:** ^1^Unit for the Diagnosis and Treatment of Congenital Metabolic Diseases, Clinical University Hospital of Santiago de Compostela, Health Research Institute of Santiago de Compostela, Santiago de Compostela, Spain; ^2^Genomes & Disease Group, Molecular Medicine and Chronic Diseases Research Centre (CiMUS), Santiago de Compostela University–IDIS, Santiago de Compostela, Spain; ^3^Molecular Genetics Unit, Clinical University Hospital of Canarias, Santa Cruz de Tenerife, Spain; ^4^Neuropediatrics Unit, Niño Jesús Clinical University Hospital, Madrid, Spain; ^5^Neuropediatrics Unit, Marqués de Valdecilla Clinical University Hospital, Santander, Spain; ^6^Genetics Unit, Marqués de Valdecilla Clinical University Hospital, Santander, Spain; ^7^Centro de Genética Médica Jacinto Magalhães, Centro Hospitalar Do Porto, Porto, Portugal; ^8^Neuropediatrics Unit, Santa María Hospital, Lisbon, Portugal; ^9^Genetics Unit, Santa María Hospital, Lisbon, Portugal; ^10^Neuropediatrics Unit, Teresa Herrera Child's Hospital, A Coruña, Spain; ^11^Clinical Genetics Unit, Teresa Herrera Child's Hospital, A Coruña, Spain; ^12^Neuropediatrics Unit, 12 de Octubre Clinical University Hospital, Madrid, Spain; ^13^Department of Medicine, Complutense University of Madrid, Madrid, Spain; ^14^Metabolic Disorders Unit, 12 de Octubre Clinical University Hospital, Madrid, Spain; ^15^Neuropediatrics Unit, Virgen de la Arrixaca Clinical University Hospital, Murcia, Spain; ^16^Genetics Unit, Clinical University Hospital of Málaga, Málaga, Spain; ^17^Neuropediatrics Unit, Clinical University Hospital of Málaga, Málaga, Spain; ^18^Neuropediatrics Unit, Clinical University Hospital Severo Ochoa, Leganés, Madrid, Spain; ^19^Clinical Genetics Unit, Pediatrics, Clinical University Hospital of Zaragoza, Zaragoza, Spain; ^20^Molecular Genetics Unit, Virgen de las Nieves Clinical University Hospital, Granada, Spain

**Keywords:** epilepsy, genetic diagnosis, neurodevelopmental disorders, *de novo* mutations, incomplete penetrance, modifier genes

## Abstract

In order to characterize the genetic architecture of epilepsy in a pediatric population from the Iberian Peninsula (including the Canary Islands), we conducted targeted exome sequencing of 246 patients with infantile-onset seizures with or without neurodevelopmental delay. We detected 107 variants in 48 different genes, which were implicated in neuronal excitability, neurodevelopment, synaptic transmission, and metabolic pathways. In 104 cases (42%) we detected variant(s) that we classified as pathogenic or likely pathogenic. Of the 48 mutated genes, 32 were dominant, 8 recessive and 8 X-linked. Of the patients for whom family studies could be performed and in whom pathogenic variants were identified in dominant or X-linked genes, 82% carried *de novo* mutations. The involvement of small copy number variations (CNVs) is 9%. The use of progressively updated custom panels with high mean vertical coverage enabled establishment of a definitive diagnosis in a large proportion of cases (42%) and detection of CNVs (even duplications) with high fidelity. In 10.5% of patients we detected associations that are pending confirmation via functional and/or familial studies. Our findings had important consequences for the clinical management of the probands, since a large proportion of the cohort had been clinically misdiagnosed, and their families were subsequently able to avail of genetic counseling. In some cases, a more appropriate treatment was selected for the patient in question, or an inappropriate treatment discontinued. Our findings suggest the existence of modifier genes that may explain the incomplete penetrance of some epilepsy-related genes. We discuss possible reasons for non-diagnosis and future research directions. Further studies will be required to uncover the roles of structural variants, epimutations, and oligogenic inheritance in epilepsy, thereby providing a more complete molecular picture of this disease. In summary, given the broad phenotypic spectrum of most epilepsy-related genes, efficient genomic tools like the targeted exome sequencing panel described here are essential for early diagnosis and treatment, and should be implemented as first-tier diagnostic tools for children with epilepsy without a clear etiologic basis.

## Introduction

Epilepsy is one of the most common neurological conditions, with a prevalence of ~1%. Etiological diagnosis of epilepsy in children using classical diagnostic tools is often a long and complex process. Moreover, most patients undergo multiple invasive and costly analyses but do not receive conclusive molecular diagnosis.

The emergence of next generation sequencing (NGS)-based tools has helped address the diagnostic challenge posed by epilepsy. These approaches allow genetic analyses to be performed faster, less expensively, and at much higher resolution. This technology has also helped identify many novel genes involved in epilepsy phenotypes.

In recent years several articles have described the use of targeted NGS or whole-exome sequencing (WES) to diagnose epileptic patients, with varying diagnostic yields. The results of these studies have helped characterize the molecular landscape of epilepsy, and indicate that infantile onset epilepsy is caused by a wide spectrum of genes, most of which are *de novo* variants in dominant genes (Epi4K Consortium et al., 2013; EuroEPINOMICS-RES Consortium, 2014; Allen et al., 2016), although a minority of recessive genes are also implicated. Mosaic mutations and copy number variants (CNVs) are other important sources of mutations in epileptic disorders (Gennaro et al., 2006; Vadlamudi et al., 2010; de Lange et al., 2017).

CNVs can cause epilepsy through the deletion or duplication of known epilepsy-related genes. Moreover, the presence of epilepsy-related genes in genomic regions affected by deletions can give rise to complex syndromic conditions (Wang et al., 2008; Dibbens et al., 2009; Helbig et al., 2009; de Kovel et al., 2010; Heinzen et al., 2010; Dejanovic et al., 2014; Epilepsy Phenome/Genome Project Epi4K Consortium, 2015; Mefford, 2015; Borlot et al., 2017; Tsuchida et al., 2018).

Recently Oates et al. (2018) demonstrated that early gene panel screening of newborns with epilepsy could reduce the cost of subsequent tests from £9,362 to £2,838 and the median diagnostic delay from 3.43 years to 21 days. Gene panel testing for epilepsy has a high diagnostic yield among children with onset before 2 years of age, and an appreciable clinical, social, and financial impact.

In cases of children with epilepsy, establishing a molecular diagnosis in a clinical setting is essential to (1) establish the risk of recurrence in subsequent pregnancies; (2) end the all-too-common diagnostic odyssey endured by parents of undiagnosed children; (3) avoid unnecessary analyses and treatments; (4) provide an accurate prognosis; (5) optimize management; (6) provide a prenatal or preimplantation diagnosis for future pregnancies; (7) identify, at least in some cases, specific appropriate therapies and enable the application of precision medicine as targeted therapeutics emerge.

The importance of genomic analyses in epilepsy has been previously discussed in depth (Lemke et al., 2012; Mercimek-Mahmutoglu et al., 2015; Mei et al., 2017; Weber et al., 2017). In this study, we present a comprehensive description of the molecular signature of pediatric epilepsy patients of Iberian origin and discuss the results obtained.

## Materials and Methods

This study was approved by the Ethics Committee of the Hospital Santiago de Compostela (Spain). A total of 246 patients were recruited over a 5-year period from different neurologic units in Spain and Portugal, applying the following inclusion criteria: patients with any condition in which seizures are either the sole clinical expression or are part of more severe neurodevelopmental disorder.

### Panel Design

Successive Epi-panels were constructed using OMIM-registered genes for which the associated clinical picture included seizures. In addition, we conducted a thorough review of the most recent scientific publications to identify genes associated with any form of epilepsy, with or without neurodevelopmental delay. We included genes for which only one published report was found, even if the involvement was suspected but not demonstrated. The selection of genes included the panel was regularly evaluated and updated throughout the study period to ensure the inclusion of all novel genes reported in the literature. Each Epi-panel included all exons and at least 25 base pairs of the flanking intronic sequence of the selected genes. A list of the genes included in the consecutive versions of each panel can be provided upon request. The first panel consisted of 88 genes and final version 274 genes. This increase reflects the high rate of discovery of epilepsy-associated genes in recent years. Because the analyses conducted at the beginning of this project were done so using comparatively incomplete panels, all undiagnosed patients who provided consent were re-analyzed at the end of the study using the most up-to-date panel in order to rule out as many false negatives as possible.

### Targeted Next Generation Sequencing

Enrichment was performed using in-solution hybridization technology (Sure Select XT; Agilent Technologies, Santa Clara, California) and subsequent sequencing using Miseq or NextSeq platforms (Illumina, Santa Clara, California), as previously described (Fernández-Marmiesse et al., 2018). Image analysis and processing of the fluorescence intensities in sequences (“base calling”) was performed with Real Time Analysis (RTA) software v.1.8.70 (Illumina), and FastQC v0.11.8 program was used for data quality control (Babraham Bioinformatics-FastQC A Quality Control Tool for High Throughput Sequence Data, 2018). Reads were aligned to the reference genome GRCh37 with BWA v0.7.17 (Li and Durbin, 2009), and BEDTools 2.27.1 (Quinlan and Hall, 2010) and Picard v2.18.14 (Picard Tools-By Broad Institute, 2018) were used for intermediate steps. VarScan v.2.4.2 (Koboldt et al., 2009), SAMtools v1.9 (Li et al., 2009), GATK v4.0.10 (McKenna et al., 2010), Pindel v0.2.5 (Ye et al., 2009), and Platypus v0.8.1 (Rimmer et al., 2014) software were used for variant detection, SnpEff v4.3 (Cingolani et al., 2012) for variant annotation, and PattRec for CNV detection (Roca et al., 2019).

Variants that passed the quality control step were prioritized according to their minor allele frequencies (MAF < 0.01) in the following databases: 1000G, Exome Aggregation Consortium (ExAC), the Exome Variant Server (EVS), the Genome Aggregation Database (gnomAD), and our in-house population database (onwards IberDB). Z-score was used to evaluate the conservation of genes which harbor rare/low-frequency variants (Roca et al., 2018). Possible pathogenicity of the missense variants detected was assessed using the *in silico* tools CONDEL (González-Pérez and López-Bigas, 2011), GERP++ (Davydov et al., 2010), and Human Splicing Finder (HSF 3.0) (Desmet et al., 2009). Variants were classified as “pathogenic” or “of uncertain significance” in accordance with the guidelines of the American College of Medical Genetics and Genomics (ACMG) (Richards et al., 2015).

### Statistical Analyses

The variants used for all calculations (unless otherwise stated) were those recorded in 2,504 individuals enrolled in Phase 3 of the 1000 Genomes Project (1000G) and in 125,748 individuals whose data are included in v2.1.1 of the Genome Aggregation Database (gnomAD). Variants classified as “pathogenic” or “likely pathogenic” in the ClinVar database were filtered out.

The mutation tolerance of each epilepsy-associated gene was quantified by determining the corresponding z-score (Roca et al., 2018). This score was calculated by first regression of the total number of missense variants against the total number of missense and synonymous variants observed for a given gene. The z-score is the corresponding studentized residual of the regression. Genes with a negative z-score have fewer missense variants than expected based on the expected mutation burden and are likely to be less tolerant of functional mutations.

To determine the probability of detecting 2 rare variants in 1 gene, or 1 rare variant in 2 genes simultaneously, we used the Poisson distribution, P(λ), for each scenario, where λ is the frequency of samples fulfilling those conditions. Only missense variants from 1000G with a MAF < 0.5% were considered.

## Results

The median (interquartile range) coverage of the samples analyzed with successive versions of the Epi-panel was 392X (309X−461X). The overall target coverage at 10X of the genes included in the successive versions of the Epi-panel ranged from 97.3 to 99.85%. However, mean target coverage increased over time, reaching 98.1–99.7% for the last 3 versions, indicating progressive optimization of the test. The regions missed were almost identical across the different samples, and shared a high GC content, repeat elements, or homology with other parts of the genome.

**Figure 1:**
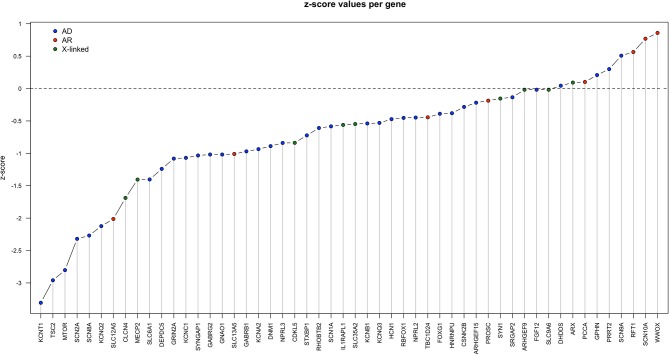
Z-score for each gene in Table 1, calculated as described in the section Material and Methods. AD, autosomal dominant inheritance; AR, autosomal recessive inheritance; X-linked: X-linked inheritance.

To prioritize the rare missense variants and assign them a higher or lower weight in terms of their contribution to the patient's phenotype we examined gene tolerance to missense variation (z-score) for all epilepsy-associated genes in Iberian patients. The results are shown in Figure 1.

**Figure 2:**
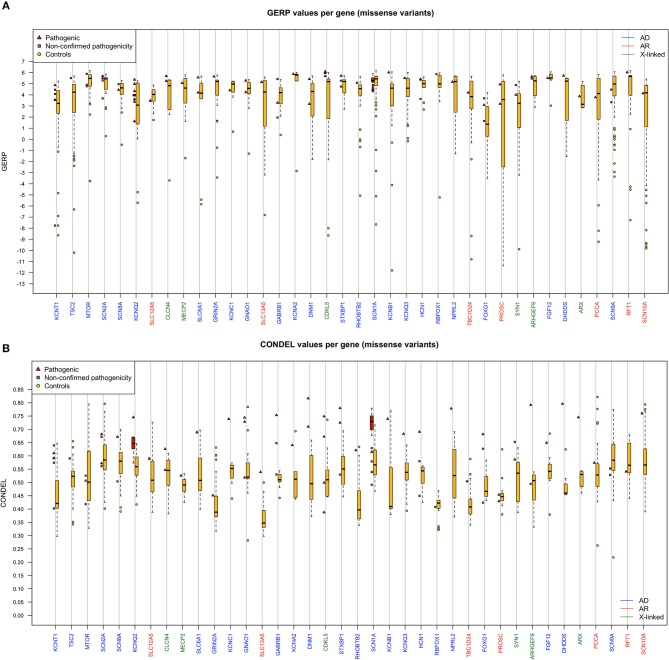
**(A,B)** Boxplot showing GERP/CONDEL scores (median and interquartile range) for missense variants. Scores for the pathogenic missense variants reported in Table 1 are shown in red, and scores for the missense variants found in control samples within each gene are shown in yellow. Only genes with pathogenic missense variants are shown. Genes with autosomal dominant inheritance (AD) are shown in blue, genes with autosomal recessive inheritance (AR) in red, and genes with X-linked inheritance in green.

Figures 2A,B show the statistical comparison of GERP and CONDEL scores for variants detected in our cohort with those of other missense variants found in the same genes in controls (extracted from 1000G and gnomAD databases). It is important to highlight that, of the missense variants found in the genes in the databases, MAF values were <0.01 in 94.88% of cases, <0.005 in 92.57% of cases, and <0.001 in 85% of cases.

**Figure 3:**
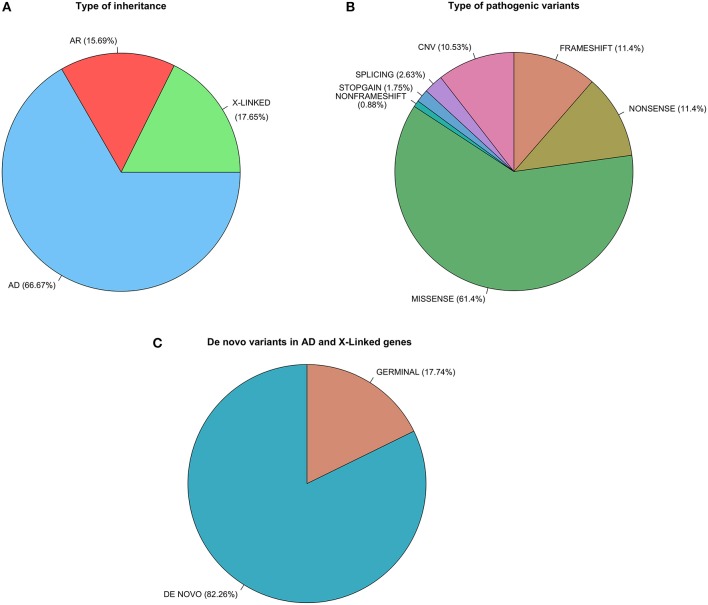
Proportions of different types of inheritance **(A)**, different types of variants **(B)**, and the proportion of *de novo* events for the Iberian epilepsy cohort **(C)**.

We detected variants in 52 different genes. Of these, 17 were involved in neuronal excitability, 30 in neurodevelopment and synaptic transmission, and 5 in metabolic pathways. Variants found in our cohort are shown in Tables 1A,B. For 32 genes (65%) variants were detected in 1 single patient, and for the following 7 genes variants were detected in more than 2 patients each: *SCN1A* (16 patients), *KCNQ2* (13 patients), *CDKL5* (7 patients), *SCN2A* (4 patients), *MECP2* (4 patients), *GNAO1* (4 patients), and *FOXG1* (3 patients). In 42% of cases the mutation identified was considered pathogenic or likely pathogenic and the main cause of patient's phenotype (Table 1A). In 10.5% of cases prioritized variants were classified as potentially involved (Table 1B), but further evidence will be required to confirm this association. It is important to note that in 9 cases variants could not be classified as pathogenic or non-pathogenic owing to a lack of variant analysis data from the progenitors required to confirm or rule out *de novo* events. Figure 3 shows the proportions of different types of inheritance and different types of variants.

**Table 1A:** Variants considered highly likely to be implicated in the patient's phenotype.

**P**	**S**	**GENE**	**INH**	**ZIG**	**VFS**	**FH**	**ClinVar**	**dbSNP ID**	**MAF**	**Type**	**ISP**	**GERP**	**Ref Seq**	**Variant**
52	F	SCN1A	AD	HT	DE NOVO	–	LP	rs121917945	–	MISS	0.73	5.29	NM_001165963.1	c.4352C>T; p.P1451L
71	F	SCN1A-2A	AD	HT	DE NOVO	–	–	–	–	CNV	-	-	NC_000002.11	g. (?_166093912)_ (167343501_?)dup
406	F	SCN1A	AD	HT	DE NOVO	–	–	–	–	NON	-	5.08	NM_001165963.1	c.2728C>T; p.Q910X
528	F	SCN1A	AD	HT	NA	–	P/LP	rs121918624	–	NON	-	5.77	NM_001165963.1	c.664C>T; p.R222X
548	F	SCN1A	AD	HT	MAT	+	CIP	rs121917922	–	MISS	0.61	5.27	NM_001165963.1	c.4973C>T; p.T1658M
597	F	SCN1A	AD	HT	DE NOVO	–	P/LP	rs794726760	–	MISS	0.76	5.27	NM_001165963.1	c.4997C>T; p.S1666F
651	M	SCN1A	AD	HT	DE NOVO	–	P/LP	rs121918624	–	NON	-	5.77	NM_001165963.1	c.664C>T; p.R222X
652	M	SCN1A	AD	HT	NA	–	P/LP	rs121917915	–	MISS	0.78	5.9	NM_001165963.1	c.4822G>T; p.D1608Y
829	M	SCN1A	AD	HT	MAT	+	–	–	–	MISS	0.68	5.79	NM_001165963.1	c.5456C>A; p.A1819E
832	M	SCN1A	AD	HT	DE NOVO	–	–	–	–	MISS	0.72	4.57	NM_001165963.1	c.C338G; p.P113R
716	F	SCN1A	AD	HT	DE NOVO	–	–	–	–	FS	-	4.9	NM_001165963.1	c.2020_2023del; p. (D674Sfs^*^15)
860	F	SCN1A		HT	DE NOVO	–	–	–	–	MISS	0.74	5.24	NM_001165963.1	c.493T>C; p.Y165H
937	M	SCN1A	AD	HT	NA	–	P	rs121917971	–	MISS	0.72	5.18	NM_001165963.1	c.2837G>A; p.R946H
980	M	SCN1A	AD	HT	NA	+	P	rs121918783	–	MISS	0.68	4.43	NM_001165963.1	c.5555T>C; p.M1852T
1112	M	SCN1A	AD	HT	PAT	+	–	–	–	MISS	0.76	5.27	NM_001165963.1	c.5009T>C; p.L1670S
1198	M	SCN1A	AD	HT	DE NOVO	–	–	–	–	MISS	0.73	5.9	NM_001165963.1	c.4658T>C; p.L1553P
314	F	SCN2A	AD	HT	NA	–	P/LP	rs796053138	–	NON	-	0.244	NM_001040142.1	c.4303C>T; p.R1435^*^
759	F	SCN2A	AD	HT	DE NOVO	–	P/LP	rs796053126	–	MISS	0.68	5.69	NM_001040142.1	c.2995G>A; p.E999K
781	M	SCN2A	AD	HT	NA	+	–	–	–	MISS	0.57	5.5	NM_001040142.1	c.G5117C; p.C1706S
1235	M	SCN2A	AD	HT	NA	–	–	–	–	NON	-	5.59	NM_001040142.1	c.2469G>A; p.W823^*^
660	M	SCN8A	AD	HT	DE NOVO	–	–	–	–	MISS	0.67	4.45	NM_014191.3	c.2620G>A; p.A874T
1173	M	SCN10A	AR	HT	NA	–	–	rs747321219	8.1E+00	FS	NA	1.16	NM_006514.3	c.5694delA; p. (A1899Qfs^*^36)
		SCN10A		HT	NA	–	–	rs774337248	1.2E+00	MISS	0.76	4.14	NM_006514.3	c.3800T>G; p.M1267R
9	F	KCNQ2	AD	HT	DE NOVO	–	P	rs864321712	-	MISS	0.65	3.89	NM_172107.3	c.319C>T; p.L107F
26	F	KCNQ2	AD	HT	DE NOVO	–	P	rs759584387	8.9E-106	MISS	0.67	1.62	NM_172107.3	c.1657C>T; p.R553W
90	M	KCNQ2	AD	HT	DE NOVO	–	CIP	rs727503974	–	MISS	0.75	4.01	NM_172107.3	c.821C>T; p.T274M
91	F	KCNQ2	AD	HT	DE NOVO	–	P	rs864321710	–	MISS	0.67	3.61	NM_172107.3	c.388G>A; p.E130K
155	F	KCNQ2	AD	HT	DE NOVO	–	LP	rs864321707	–	MISS	0.65	4.01	NM_172107.3	c.917C>T; p.A306V
158	M	KCNQ2	AD	HT	DE NOVO	–	P	rs864321706	–	MISS	0.63	4.01	NM_172107.3	c.850T>G; p.Y284D
475	F	KCNQ2	AD	HT	DE NOVO	–	–	–	–	MISS	0.57	3.38	NM_172107.3	c.778C>T; p.H260Y
485	F	KCNQ2	AD	HT	NA	–	–	–	–	MISS	0.65	4.01	NM_172107.3	c.829A>T; p.T277S
571	M	KCNQ2	AD	HT	DE NOVO	–	P	rs886041262	–	MISS	0.60	4.05	NM_172107.3	c.629G>A; p.R210H
704	F	KCNQ2	AD	HT	DE NOVO	–	LP	rs1057523728	–	MISS	0.58	4.01	NM_172107.3	c.833T>C; p.I278T
744	F	KCNQ2	AD	HT	NA	–	LP	rs897976020	1.23E-105	MISS	0.64	5.37	NM_172107.3	c.1588G>A; p.E530K
875	M	KCNQ2	AD	HT	PAT	+	–	–	–	MISS	0.64	4.34	NM_172107.3	c.1016T>A; p.L339Q
1085	M	KCNQ2	AD	HT	DE NOVO	–	P	rs118192234	–	MISS	0.67	4.99	NM_172107.3	c.1658G>A; p.R553Q
84	M	KCNQ3	AD	HT	NA	+	–	–	–	MISS	0.68	5.51	NM_004519.3	c.899T>C; p.F300S
868	F	KCNA2	AD	HT	DE NOVO	–	–	–	–	MISS	0.64	5.87	NM_004974.3	c.959C>T; p.T320I
109	M	KCNB1	AD	HT	DE NOVO	–	–	–	–	MISS	0.74	6.02	NM_004975.3	c.1107G>C; p.W369C
41	M	KCNT1	AD	HT	TRANS	–	–	–	–	SP	-	5.24	NM_020822.2	c.3503-1G>A
		KCNT1	AD			–	P	–	–	MISS	0.61	4.87	NM_020822.2	c.785G>A; p.R262Q
957	M	KCNT1	AD	HT	NA	–	P	–	–	MISS	0.61	4.87	NM_020822.2	c.785G>A; p. R262Q
1068	M	KCNC1	AD	HT	NA	–	–	–	–	MISS	0.74	4.41	NM_001112741.1	c.88C>T; p.P30S
501	M	SLC12A5	AR	HO	TRANS	–	–	–	–	MISS	0.59	3.47	NM_001134771.1	c.3274G>A; p.E1092K
901	M	CLCN4	XL	HE	MAT	+	–	–	–	MISS	0.55	5.69	NM_001830.3	c.1576G>A; p.G526S
1177	M	CLCN4	XL	HE	NA	–	–	–	–	MISS	0.62	5.25	NM_001830.3	c.1597G>A; p.V533M
658	M	HCN1	AD	HT	DE NOVO	–	LP	rs1057519547	–	MISS	0.69	5.42	NM_021072.3	c.1172G>A; p.G391D
67	M	SLC13A5	AR	HT	TRANS	–	–	rs1211773372	–	FS	-	3.12	NM_177550.4	c.1511delT; p. (L504Cfs^*^23)
		SLC13A5		HT		–	–	rs936922976	–	MISS	0.54	5.15	NM_177550.4	c.1514C>T; p.P505L
1030	F	FGF12	AD	HT	NA	–	P	rs886039903	–	MISS	0.65	5.51	NM_021032.4	c.341G>A; p.R114H
37	F	PRRT2	AD	HT	NA	+		–	–	CNV	-	-	NC_000016.10	g. (?_29824300)_ (29826034_?)del
207 629 975, 1129	F	PRRT2	AD	HT	NA	+	P	rs587778771	0.007	FS	-	3.9	NM_001256442.1	c.649_650insC; p. (R217Pfs^*^8)
545	F	ARX	XL	HT	DE NOVO	–	–	–	–	MISS	0.74	3.87	NM_139058.2	c.1039T>G; p.F347V
385	M	MECP2	XL	HE	DE NOVO	–	–	–	–	NON	-	5.67	NM_001110792.1	c.1386C>G; p.Y462X
474	F	MECP2	XL	HT	NA	–	P	rs886041728	–	FS	-	5.11	NM_001110792.1	c.1087_1088insC; p. (K363Tfs^*^30)
572	M	MECP2	XL	HE	NA	–	P/LP	rs61751443	–	MISS	0.52	5.06	NM_001110792.1	c.917G>A; p.R306H
965	F	MECP2	XL	HT	NA	–	–	–	–	NON	-	5.8	NM_001110792.1	c.1453G>T; p.E485^*^
420	F	FOXG1	AD	HT	NA	–	–	–	–	NON	-	4.05	NM_005249.4	c.764G>A; p.W255X
427	M	FOXG1	AD	HT	DE NOVO	–	P/LP	rs398124202	-	NON	-	2.03	NM_005249.4	c.256C>T; p.Q86X
872	M	FOXG1	AD	HT	DE NOVO	–	–	–	–	MISS	0.68	3.09	NM_005249.4	c.565C>T; p.L189F
47	F	CDKL5	XL	HT	DE NOVO	–	–	–	–	MISS	0.67	5.69	NM_001037343.1	c.176G>C; p.R59P
131	F	CDKL5	XL	HT	DE NOVO	–	–	–	–	MISS	0.50	6.07	NM_001037343.1	c.455G>A; p.C152Y
156	F	CDKL5	XL	HT	DE NOVO	–	–	–	–	FS	-	−0.27	NM_001037343.1	c.2635_2636del; p. (L879Efs^*^30)
202	F	CDKL5	XL	HT	DE NOVO	–	LP	rs267608433	–	FS	-	4.59	NM_001037343.1	c.163_166del; p. (E55Rfs^*^20)
472	M	CDKL5	XL	HE	DE NOVO	–	P/LP	rs267608659	–	NON	-	6.03	NM_001037343.1	c.2413C>T; p.Q805X
473	F	CDKL5	XL	HT	DE NOVO	–	–	–	–	MISS	0.39	6.1	NM_001037343.1	c.616G>T; p.E206Y
1127	F	CDKL5	XL	HT	NA	–	–	–	–	MISS	0.75	5.98	NM_001037343.1	c.380A>T; p.H127L
310	M	HNRNPU	AD	HT	DE NOVO	–	–	–	–	FS	-	4.34	NM_031844.2	c.401_402delAC; p. (D134Gfs^*^10)
421	M	TBC1D24	AR	HO	TRANS	–	P/LP	rs398122965	1.5E-105	MISS	0.50	4.2	NM_001199107.1	c.724C>T; p.R242C
317	F	GNAO1	AD	HT	DE NOVO	–	P	rs587777057	–	MISS	0.73	5.28	NM_020988.2	c.607G>A; p.G203R
684	F	GNAO1	AD	HT	DE NOVO	–	P	rs1064794533	–	MISS	0.74	5.29	NM_020988.2	c.709G>A; p.D237L
1208	F	GNAO1	AD	HT	DE NOVO	–	P	rs587777057	–	MISS	0.73	5.28	NM_020988.2	c.607G>A; p.G203R
606	M	GNAO1	AD	HT	NA	–	–	–	–	MISS	0.52	4.24	NM_138736.2	c.901G>C; p.V301L
289	M	CSNK2B	AD	HT	DE NOVO	–	–	rs1339457069	0	NON	-	4.88	NM_001320.6	c.124C>T; p.Gln42^*^
164	M	SLC9A6	XL	HE	DE NOVO	–	–	–	–	NON	-	5.36	NM_001042537.1	c.1601C>G; p.S534^*^
1238	M	WWOX	AR	HT	TRANS	–	–	–	–	NON	-	-	NM_016373.3	c.1114G>T; p.G372^*^
		WWOX		HT		–	–	–	–	CNV	-	-	NG_011698.1 (NM_016373.3)	c. (605+1_606-1)_(791+1_792-1)del
996	M	NPRL2	AD	HT	PAT	+	–	–	–	MISS	0.78	5.17	NM_006545.4	c.949G>A; p.G317R
1213	M	NPRL3	AD	HT	NA	+	–	–	–	NON	NA		NM_001077350.2	c.138delC; p.W46^*^
951	M	DEPDC5	AD	HT	EN CURSO	–	–	–	–	CNV	-	-	NG_034067.1 (NM_001242896.1)	c. (1081+1_1082-1)_(1143+1_1144-1)del
782	M	SRGAP2	AD	HT	NA	–	–	–	–	CNV	-	-	NG_033804.1 (NM_015326.4)	c. (?_0)_(570+1_571-1)dupc. (897+1_898-1)_(^*^3018_?)dup
519	M	RBFOX1	AD	HT	PAT	–	–	–	–	CNV	-	-	NG_011881.1 (NM_001308117.1)	c. (3+1_4-1)_(114+1_115-1)del
530	M	GPHN	AD		MAT	–	–	–	–	CNV	-	-	NG_008875.1 (NM_020806.4)	c. (143+1_144-1)_(294+1_295-1)del
819	M	GPHN	AD	HT	MAT	–	–	–	–	CNV	-	-	NG_008875.1 (NM_020806.4)	c. (143+1_144-1)_(^*^871_?)del
186	F	ARHGEF9	XL	HT	DE NOVO	–	–	–	–	MISS	0.79	5.65	NM_015185.2	c.541G>C; p.G181R
1126	M	SLC6A1	AD	HT	NA	–	–	–	–	MISS	0.69	4.21	NM_003042.3	c.373G>A; p.V125M
1156	F	SLC6A1	AD	HT	DE NOVO	–	–	–	–	MISS	0.69	5.57	NM_003042.3	c.1153T>C; p.F385L
1058	M	IL1RAPL1	XL	MOS	DE NOVO	–	–	–	–	CNV	-	-	NG_008292.1 (NM_014271.3)	c. (?_−24)_(1057+1_1058-1)del
598	M	STXBP1	AD	HT	DE NOVO	–	P/LP	rs796053353	–	MISS	0.72	5.71	NM_003165.3	c.416C>T; p.P139L
1214	M	STXBP1	AD	HT	NA	–	P	rs796053361	–	MISS	0.78	4.75	NM_003165.3	c.875G>A; p.R292H
1233	F	STXBP1	AD	HT	DE NOVO	–	–	–	–	SPL	NA	5.62	NM_003165.3	c.1548-2A>G
201	F	DNM1	AD	HT	DE NOVO	–	US	rs751244420	5.4E-105	MISS	0.50	1.83	NM_025014.1	c.112G>A; p.G38S
481	M	DNM1	AD	HT	DE NOVO	–	–	–	–	MISS	0.71	5.42	NM_001288739.1	c.442C>A; p.Q148L
740	M	SYN1	XL	HE	MAT	+	–	–	–	MISS	0.59	4.01	NM_006950.3	c.376T>A; p.W126R
218	F	GRIN2A	AD	HT	DE NOVO	–	–	rs1445802934	8.9E-106	MISS	0.45	5.65	NM_000833.4	c.2069C>T; p.T690M
857	F	ARHGEF15	AD	HT	NA	–	–	–	–	NONFS	-	−0.65	NM_025014.1	c.709_723del; p.Val237_Ala241del
685	M	RHOBTB2	AD	HT	DE NOVO	–	–	–	–	MISS	0.62	5.08	NM_001160036.1	c.1531C>T; p.R511W
904	M	GABRG2	AD	HT	PAT	+	–	–	–	NON	-	5.56	NM_198903.2	c.824G>A; p.W275^*^
76	M	SYNGAP1	AD	HT	DE NOVO	–	–	–	–	NON	-	4.8	NM_006772.2	c.1264G>T; p.E422^*^
859	F	SLC35A2	AD	HT	NA	–	–	–	–	FS	-	4.27	NM_001282651.1	c.636_657del; p. (Q213Hfs^*^157)
561	M	RFT1	AR	HO	TRANS	–	US	rs148716754	0.003	MISS	0.54	6.03	NM_052859.3	c.47C>G; p.S16C
1197	M	DHDDS	AR,AD	HT	DE NOVO	–	–	–	–	MISS	0.79	5.72	NM_024887.3	c.110G>A; p.R37H
954	M	PROSC	AR	HT	TRANS	–	–	rs79148472	0.005	MISS	0.43	3.19	NM_007198.3	c.157A>G; p.M53V
		PROSC		HT		–	–	rs150307985	9.85E-105	MISS	0.40	4.94	NM_007198.3	c.445G>A; p.G149R
38	M	PCCA	AR	HT	TRANS	–	–	–	–	SP	-	2.48	NM_000282.3	c.469-4A>G
		PCCA		HT		–	–	rs146927771	0.0012	MISS	0.57	3.78	NM_000282.3	c.929C>G; p.A310G

*P, patient; S, sex; M, male; F, female; INH, type of inheritance described for the gene up to now; AD, autosomal dominant; AR, autosomal recessive; XL, X-linked; ZYG, zygosity; HT, heterozygous; HO, homozygous; HE, hemizygous; VFS, variant family study; M, maternal; PAT, paternal; Abbreviations: NA, not available; FH, familial history; ClinVar, classification in the variant in ClinVar database; P, pathogenic; LP, likely pathogenic; US, uncertain significance; B, benign; LB, likely benign; dbSNP, ID in Single Nucleotide Polymorphism Database; MAF, minor allele frequency in public databases; TYPE, functional consequence; MISS, missense; NON, nonsense, SP, splicing; FS, frameshift; NONFS, nonframeshift; CNV, copy number variant; GERP, genomic evolutionary rate profiling score; ISP, in silico prediction with CONDEL software; REFSEQ, Reference Sequence*.

**Table 1B:** Variants of uncertain significance [further studies required to confirm relationship between variant(s) and patient's phenotype].

**P**	**S**	**GENE**	**INH**	**ZYG**	**VFS**	**FH**	**ClinVar**	**dbSNP**	**MAF**	**TYPE**	**ISP**	**GERP**	**Ref seq**	**Variant**
**FUNCTIONAL VALIDATION REQUIRED**														
248	F	SCN9A	AD	HT	TRANS	–	–	rs180949263	0.0002	MISS	0.55	3.34	NM_002977.3	c.5672G>A/p.R1891H
		SCN9A	AD	HT		–	B/LB	rs202084411	0.0024	MISS	0.53	4.47	NM_002977.3	c.4612T>C/p.W1538R
		CHD2	AD	HT	MAT	–	–	–	–	MISS	0.53	5.69	NM_001271.3	c.4564G>A/p.D1522N
918	M	MTOR	AD	HT	PAT	–	–	rs1390645065	8.9E-106	MISS	0.52	5.89	NM_004958.3	c.7249G>A/p.V2417M
		MTOR	AD	HT	MAT	–	–	rs201557303	0.0002	MISS	0.42	4.91	NM_004958.3	c.5197G>A/p.A1733T
1084	M	MTOR	AD	HT	PAT	–	–	–	–	MISS	0.51	4.78	NM_004958.3	c.126G>T/p.K42N
1144	M	TSC2	AD	HT	PAT	–	–	–	–	MISS	0.59	5.51	NM_000548.4	c.1724T>C/p.L575P
**INCOMPLETE PENETRANCE**														
754	M	GABRB1	AD	HT	MAT	–	LP	rs1135401786	–	MISS	0.75	3.29	NM_000812.3	c.157C>T; p.R53W
938	F	GABRB1	AD	HT	MAT	–	–	–	–	MISS	0.53	5.43	NM_000812.3	c.775A>G/p.I259V
703	M	SCN2A	AD	HT	PAT	–	–	–	–	MISS	0.67	3.68	NM_001040142.1	c.5551C>T/p.R1851W
801	F	SCN1A	AD	HT	MAT	–	US	rs184524479	0.00019	MISS	0.49	4.33	NM_001165963.1	c.1604G>A/p.R535H
		RBFOX1	AD	HT	MAT	–	–	rs1287710352	8.9E-106	MISS	0.41	5.86	NM_145891.2	c.983A>G/p.K328R
1119	F	SYN1	XL	HT	PAT	–	–	–	–	MISS	0.65	4.88	NM_006950.3	c.718G>A/p.G240R
650	M	FOXG1	AD	HT	PAT	–	–	–	–	MISS	0.63	3.69	NM_005249.4	c.655C>G/p.R219G
1008	M	KCNT1	AD	HT	MAT	–	–	–	–	MISS	NA	4.46	NM_020822.2	c.2912_2913delinsAT/p. (S971N)
268	M	KCNT1	AD	HT	PAT	–	–	–	–	MISS	0.59	3.54	NM_020822.2	c.1421G>T/p.R474L
**PLAUSIBLE OLIGOGENIC INHERITANCE**														
13	M	SCN1A	AD	HT	MAT	–	B/LB	rs121917956	0.002	MISS	0.58	4.89	NM_001165963.1	c.5749C>G/p.R1917G
		CLCN2	AR,AD		PAT	–	LB	rs61729156	0.0068	MISS	0.53	2.83	NM_004366.5	c.203G>A/p.R68H
141	F	SCN1A	AD	HT	PAT	–	-	rs1226308717	-	MISS	0.54	5.44	NM_001165963.1	c.3376A>G/p.N1126D
		SCN1A	AD	HT	PAT	–	B/LB	rs121917956	0.002	MISS	0.58	4.89	NM_001165963.1	c.5782C>G/p.R1928G
		KCNT1	AD	HT	MAT	–	LB	rs148162797	0.0008	MISS	0.57	−7.76	NM_020822.2	c.2220C>G/p.D740E
396	M	SCN1A	AD	HT	NA	–	B/LB	rs121917956	0.002	MISS	0.58	4.89	NM_001165963.1	c.5749C>G/p.R1917G
		CLCN2	AD	HT	NA	–	–	rs202031742	1.5E-105	MISS	0.47	4.07	NM_004366.5	c.368C>T/p.S123F
1113	M	HCN1	AD	HT	NA	–	–	rs1462729387	4.1E-106	MISS	0.45	3.63	NM_021072.3	c.2269A>G/p.T757A
		KCNT1	AD	HT	NA	–	CIP	rs201156458	0.00028	MISS	0.40	4.08	NM_020822.2	c.3256G>A/p.G1086R
		CACNA1H	AD	HT	NA	–	LB	rs187596702	0.00040	MISS	0.49	3.52	NM_021098.2	c.385G>A/p.G129S
968	F	SCN1A	AD	HT	MAT	–	–	–	–	MISS	0.63	5.18	NM_001165963.1	c.2785C>T/ p.L929F
		SLC12A5	AD	HT	NA	–	–	–	–	MISS	0.59	3.47	NM_001134771.1	c.3343G>A/ p.E1115K
		CACNA1A	AD	HT	PAT	–	US	rs200850308	8.2e-05	MISS	0.56	5.19	NM_023035.2	c.3179G>A
379	F	SCN2A	AD	HT	NA	–	CIP	rs149987700	0.0003	MISS	0.56	5.29	NM_001040142.1	c.952G>A/p.E318K
		SCN7A				–	–	rs146072866	0.001	MISS	0.58	−0.257	NM_002976.3	c.2431A>G/p.S811G
**DATA FROM PROGENITORS UNAVAILABLE:** ***DE NOVO*** **MUTATION STATUS UNCONFIRMED**														
1038	M	SCN8A	AD	HT	NA	+	–	–	–	MISS	0.50	4.96	NM_014191.3	c.4633A>C/ p.T1545P
981		HCN1	AD	HT	NA	–	–	–	–	MISS	0.58	5.37	NM_021072.3	c.715G>T p.V239L
738	F	FOXG1	AD	HT	NA	–	–	–	–	MISS	0.42	1.65	NM_005249.4	c.458G>T/p.G153V
887	M	SCN1A	AD	HT	NA	–	–	rs777120925	7.49e-05	MISS	0.54	4.75	NM_001165963.1	c.1457C>G/p.A486G
		CHRNA4	AD	HT	NA	–	–	–	–	MISS	0.69	5.06	NM_000744.6	c.803C>T/p.P268L
523	F	STXBP1	AD	HT	NA	–	P	rs746172968	8,2E-106	MISS	0.53	5.27	NM_003165.3	c.1756G>A/p.D586N
		SIK1	AD	HT	NA	–	–	–	–	MISS	0.59	3.93	NM_001320643.1	c.1829G>Tp.R610L
676	M	HECW2	AD	HT	NA	–	–	–	–	MISS	0.46	2.43	NM_020760.2	c.3939C>G/p.I1313M
		GABRB1	AD	HT	NA	–	–	–	–	MISS	0.65	1.96	NM_000812.3	c.1017G>C/p.K339N
		GABBR2	AD	HT	NA	–	–	–	–	MISS	0.68	5.64	NM_005458.7	c.1858C>A/p.P620T
1029	M	GRIA3	XLR	HE	NA	–	–	–	–	MISS	0.41	5.52	NM_000828.4	c.793G>A/p.A265T
1099	M	HECW2	AD	HT	NA	–	–	–	–	MISS	0.44	5.10	NM_020760.2	c.900C>A/p.S300R
884	F	ARHGEF9	XL	HT	NA	–	–	–	–	MISS	0.49	5.46	NM_015185.2	c.1454G>T/p.G485V

**Figure 4:**
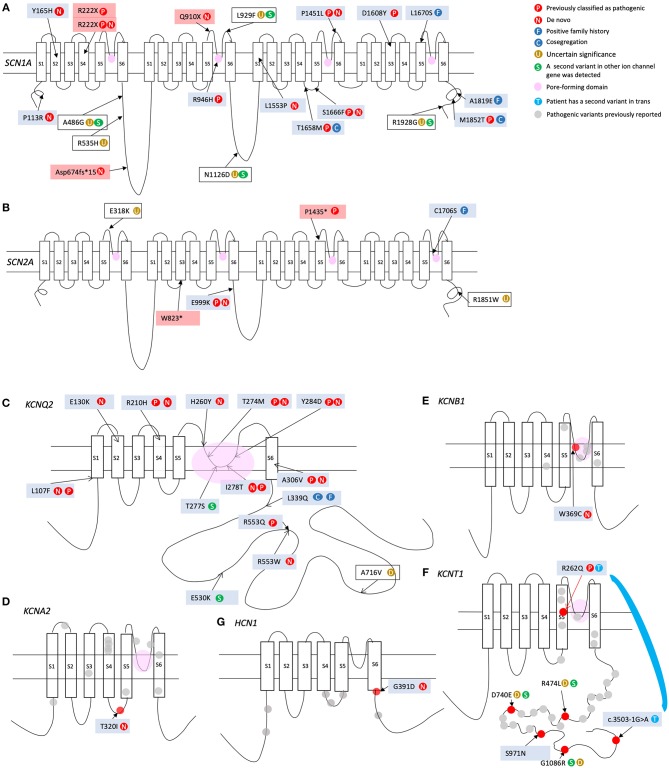
Locations of variants found in our cohort in the proteins encoded by the following genes: **(A)** *SCN1A*; **(B)** *SCN2A*; **(C)** *KCNQ2*; **(D)** *KCNA2*; **(E)** *KCNB1*; **(F)** *KCNT1*; **(G)** *HCN1*.

**Figure 5:**
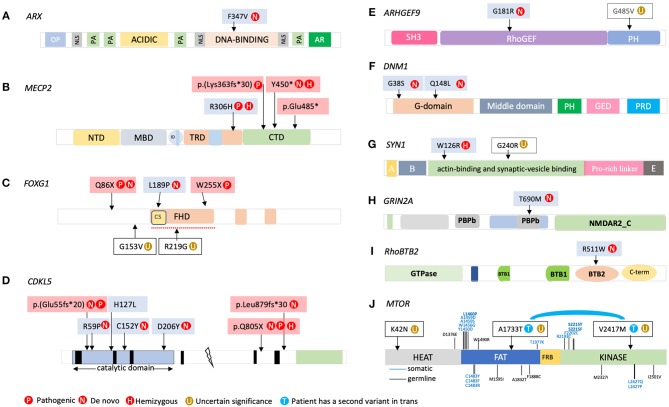
Locations of variants found in our cohort in the following genes: **(A)** *ARX*; **(B)** *MECP2*; **(C)** *FOXG1*; **(D)** *CDKL5*; **(E)** *ARHGEF9*; **(F)** *DNM1*; **(G)** *SYN1*; **(H)** *GRIN2A*; **(I)** *RhoBTB2*; **(J)** *MTOR*. NTD, N-terminal domain; MBD, methyl CpG binding domain; TRD, transcriptional repression domain; NLS, nuclear localization signal; CTD, C-terminal domain; FHD, forkhead domain.

**Figure 6:**
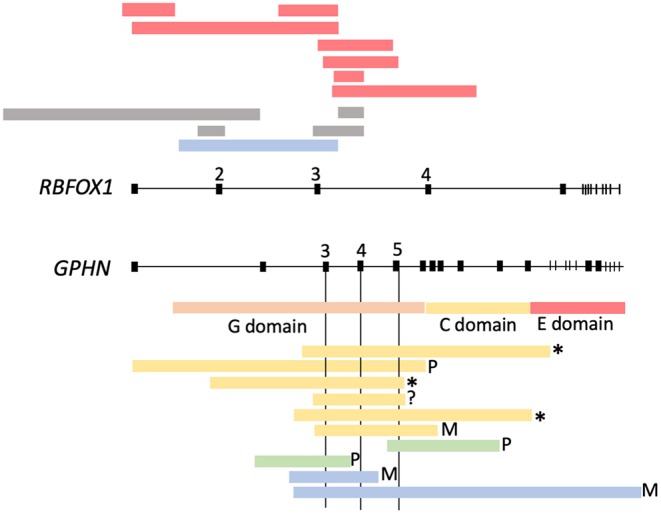
Microdeletions found in *RBFOX1* and *GPHN*. Yellow, (Lionel et al., 2013); green, (Dejanovic et al., 2014); blue, Fernández-Marmiesse (present study).

The clinical features of the patients carrying the variants that appear in Tables 1A,B are listed in the Supplementary Table 1. The locations of the variants in some of the genes are shown in Figures 4, 5. The position of 2 CNVs in *FOXG1* and *GPHN* relative to those previously reported is shown in Figure 6.

## Discussion

### Utility of Z-Score and GERP and CONDEL Scores for Variant Prioritization

We found that z-score and GERP and CONDEL scores can be combined to provide an effective means of assessing the potential pathogenicity of variants detected in epilepsy patients.

A negative z-score implies a marked sensitivity to missense variation, and indicates that missense variations in these genes are much more likely to affect the function of the encoded proteins. The genes most sensitive to missense variation were *KCNT1, TSC2*, and *MTOR* (Figure 1). However, the variants detected in these 3 genes in our cohort were classified as “of uncertain significance,” as explained below. The next most sensitive genes were 3 ion-channel-encoding genes (*SCN2A, SCN8A, KCNQ2*), mutations in which are found in a significant percentage of patients with neurodevelopmental problems. A higher z-score indicates a lower sensitivity to missense variation. For example, *WWOX* (z-score > 3) is a recessive gene with low sensitivity to missense changes. In fact, most disease-associated mutations described for this gene are truncating mutations (Shaukat et al., 2018). *SCN10A* and *RFT1* are recessive genes and therefore had the highest z-scores.

As shown in Figures 2A,B, GERP and CONDEL scores constitute a useful means of discriminating between innocuous and deleterious variants. For some highly conserved genes (*MTOR, SCN2A, SCN8A, KCNA2, SCN1A, DNM1, KCNC1, GNAO1, STXBP1, GRIN2A, HCN1, NPRL2*), we observed very high mean GERP values and low variability (Figure 2A). Any missense variation in these genes can be considered potentially deleterious. For other genes (*KCNQ2, SLC13A5, KCNT1, PROSC*) the range of GERP scores was much wider, implying that some nucleotides are much more sensitive to variation than others, and therefore that in addition to CONDEL score the location of the variant is important.

As shown in Figure 2B, CONDEL score allowed effective discrimination between deleterious and innocuous variants for the 2 most mutated genes in our cohort: *KCNQ2* and *SCN1A* (*p* = 2.8e^−5^ and 1.12e^−6^, respectively; Wilcoxon test). In general, for all variants shown in Table 1A, CONDEL scores were above the upper limit of distribution of the control variants (yellow bars), except for 2 variants in *RFT1* (recessive) and *CDKL5* (X-linked). Interestingly, both these variants had very high GERP scores. This suggests that even though the amino acid change is functionally relatively innocuous, its location may have a significant impact on the protein (e.g., from regulatory point of view), since it is highly conserved throughout evolution.

### Variants Classified as Pathogenic and Likely Pathogenic

#### Most Mutated Genes

We detected 23 variants in *SCN1A* (Tables 1A,B and Figure 3A), including a long duplication that encompassed the sodium channel cluster (2q24.3) and was confirmed by array comparative genomic hybridization (CGH). Of the 23 *SCN1A* variants detected 16 were considered highly likely to contribute to the patient's phenotype (Table 1A). As previously noted in other studies, missense variants in this gene were associated with milder phenotypes, except for those located in the pore region of the channel. We also found that phenotype severity was inversely proportional to familial incidence (Meng et al., 2015).

Six patients carried *SCN2A* variants, but only 4 were classified as pathogenic or likely pathogenic (Figure 4B). As expected, the 2 *SCN2A* truncating variants were associated with a late-onset ASD/ID phenotype that was unresponsive to Na^+^ channel blockers. Conversely, the missense variant in P759 behaved as a gain of function (GOF) variant and was associated with early onset and a severe seizure phenotype.

We detected 13 missense *KCNQ2* variants in our cohort, 6 of the which had been previously described by our group in an article providing extensive information on phenotype-genotype relationship (Hortigüela et al., 2017). As expected, the phenotypes of patients with variants located in the pore-forming domain were severe (Figure 4C).

Seven patients from our cohort carried variants in *KCNT1*. The lower z-score of this gene (Figure 1) indicates high sensitivity to missense variation. However, owing to the variable penetrance of variants in this gene it is difficult to demonstrate a definitive association between these variants and the patients' phenotypes. Patient P41 was found to carry biallelic *KCNT1* mutations; one caused altered splicing and the other was a missense variant (R262Q) previously described as a *de novo* variant in an individual with malignant migrating focal seizures of infancy. In line with the variable penetrance previously reported for this gene, the progenitor of the patient carrying this mutation (P41) was healthy. This is not the first case of recessive inheritance in this gene (Martin et al., 2014; Møller et al., 2015). The same missense pathogenic variant R262Q was detected in P957. However, in this case it was not possible to demonstrate a *de novo* event. While another missense heterozygous indel leading to the missense change S971N was detected in P1008, a familial study could not be performed for this particular patient, preventing classification of the variant. Finally, variant R474L was detected in P268. This variant was found to be paternally inherited. Nonetheless, we consider it highly likely that this variant contributes to the patient's phenotype, as 3 variants affecting the same amino acid (R474S, R474C, and R474H) are classified as disease-associated variants in the ClinVar database (Barcia et al., 2012). The incomplete penetrance previously described for this gene (Møller et al., 2015) remains to be explained.

Five variants were detected in *FOXG1*. Of the 3 missense variants, 2 were located in the conserved forkhead domain (FHD; Figure 5C) where most disease-associated missense variants have been found (Mitter et al., 2018). R219G was a paternally inherited variant located in this key region. We observed high GERP and CONDEL scores for this variant. However, the clinical phenotype did not match that typically associated with *FOXG1* mutations (no microcephaly, no stereotypical movements, and no regression) and therefore it was classified as of uncertain significance. Similarly, variant G153V was not located in the DNA-binding forkhead domain, *in-silico* predictions did not support its deleteriousness, and the patient's clinical picture did not match that typically associated with FOXG1 mutations.

We detected 7 variants in *CDKL5* (Figure 5D). The 4 missense *CDKL5* mutations were located in the N-terminal kinase domain, which plays a particularly important role in brain function (Fehr et al., 2013). All were the result of *de novo* mutations, except for the variant detected in P1127, for which we observed very high GERP and CONDEL scores and an associated phenotype concordant with this disorder.

Four patients carried heterozygous missense variants in *GNAO1. Two* of these patients carried the same variant. Our analyses revealed that this gene was one of the best conserved, with a low z-score (Figure 1) and a mean GERP score > 5 (Figure 2A). The phenotype of these 4 patients perfectly matched that described for *GNAO1*-mutation carriers. Unfortunately, it was not possible to test the *de novo* status of variant V301L in P606. This variant is located in a highly conserved nucleotide. Because the phenotype fitted well with that described for *GNAO1* mutations, we classified this variant as likely pathogenic, although familiar or functional studies will be required to support this classification.

#### Variants With Special Characteristics

P186 and P884 carried a heterozygous missense variant in the X-linked gene *ARHGEF9*. In P186, the variant was located in the RhoGEF domain, which encodes the guanine nucleotide exchange factor (GEF) activity of collybistin, while in P884 the variant was located in the C-terminal domain (Figure 5E). While the CONDEL score for this variant was low, Human Splicing Finder software revealed that this nucleotide change promotes the emergence of a cryptic donor splice site ~65% stronger than the wild-type site, potentially affecting the splicing of the last exon. Due to a lack of patient RNA sample and samples from family members it was not possible to determine the true involvement of this variant in the patient's phenotype.

P685 carried a *de novo* missense variant in the BTB2 domain of *RHOBTB2* (Figure 5I). This variant was located in an amino acid contiguous to that in which a *de novo* missense variant was previously described in a female patient (Lopes et al., 2016) who exhibited developmental stagnation at 6–9 months, coinciding with the onset of generalized epilepsy and additional clinical signs including Rett syndrome-like hand stereotypies, intense eye communication, and sleep problems.

#### CNVs Detected in Our Epilepsy Cohort

As mentioned in the introduction, genomic CNVs account for a substantial proportion of the genetic burden in about 3% of patients with idiopathic epilepsies, and increase the risk of idiopathic generalized epilepsy and a wide range of neurodevelopmental disorders (Helbig et al., 2009; de Kovel et al., 2010; Heinzen et al., 2010; Coe et al., 2012; Lal et al., 2013; Møller et al., 2013). We detected 9 CNVs in our cohort. One microdeletion in *RBFOX1* encompassing exons 2 and 3 (Δ2+3) was detected in P519. Figure 6 shows several of the microdeletions reported for this gene. All affect exons at the 5′ end of the gene. Some are proven *de novo* mutations, while others are inherited from an unaffected progenitor, indicating variable penetrance. We found 2 microdeletions in *GPHN* in patients P530 and P819, both of which were maternally inherited. Deletions affecting *GPHN* exons are extremely rare in the general population (Mefford et al., 2011; González et al., 2013; Lionel et al., 2013; Dejanovic et al., 2014). Previously described microdeletions in this gene are summarized in Figure 6. The common overlapping region across the 10 deletions encompasses exons 3 and/or 5, which encode the G domain of the gephyrin protein. G-domain trimerization is vital for the formation of the hexagonal gephyrin oligomer scaffolds required for stable GABA receptor clustering in postsynaptic inhibitory neurons.

### Variants of Unknown Significance

#### Variants for Which Additional Evidence Is Required to Support Classification as Deleterious: Lack of Familial Studies and/or Functional Validation

P248 presented secondary partial epilepsy that was unresponsive to treatment, PMD, and Rett syndrome-like features, and was found to carry 2 biallelic mutations in *SCN9A*. In this case it would be necessary to demonstrate that the combined effects of 2 variants in opposite alleles give rise to the clinical phenotype. This is the first case in which biallelic mutations in *SCN9A* have been described.

In one (P523) of the 4 patients who carried variants in *STXBP1* we detected a second missense variant in *SIK1* (Table 1B). *In silico* analysis predicted that both variants were likely deleterious to the encoded protein. Unfortunately, it was not possible to determine the contribution of these 2 variants to the patient's phenotype owing to the absence of DNA samples and clinical histories for the patient's parents.

##### The uncertain significance of the variant in TSC2

In P1144, a male with neonatal seizures characteristic of tuberous sclerosis (TSC) who responded well to vigabatrin and is progressing toward normality, we detected a missense variant in *TSC2*. The very high GERP and CONDEL scores for this gene indicate a high degree of sensitivity to variation (this gene had the second lowest z-score of all genes studied). These findings support a functional impact of this variant. While the majority of TSC patients are diagnosed during the first 15 months of life, the disease often goes unnoticed owing to the wide phenotypic variability. Familial cases of TSC are caused by germline mutations, but 70% of cases are the result of somatic mutations (Lim et al., 2017). Familial transmission results in mild-to-moderate disease that may not meet all diagnostic criteria. In most such cases the first clinical sign is seizures. All types of seizures can be observed in TSC patients. Two thirds of cases begin with focal refractory epilepsy. TSC patients have an increased risk of other neurocognitive deficits, including ASD, ID, and mood alterations. A response to vigabatrin is characteristic of these patients, but not distinctive. P1144 carried a paternally inherited missense variant (L575P) in *TSC2*. We received a blood sample from this patient a few days after her birth and the discovery of the variant surprised her pediatrician, since the patient's seizures closely resembled those of TSC patients. However, to date no cutaneous stigmas or tubers have been detected. The patient's father, who also carries the mutation, underwent magnetic resonance imaging (MRI) but no tubers were detected. P1144 has 2 siblings, both of whom were positive for the *TSC2* variant. Neither of the siblings display clinical signs characteristic of TSC, but one is under study for short height with no apparent cause and the other was born with unilateral double ureter with grade IV reflux. MRI has been prescribed for both siblings. Recent studies (Caylor et al., 2018; Liu et al., 2018) have identified cases of *TSC2* mutations in which the initial presentation consisted of seizures and describe the incidental diagnosis of asymptomatic family members. Further studies and follow-up are required to confirm the involvement of this variant in the phenotypes of the family members. Given the importance of a timely TSC diagnosis for appropriate clinical management, these cases highlight the potential benefits of an unbiased molecular diagnostic approach.

##### Variants in genes involved inTOR and GATOR complexes

Six of our patients carried variants in genes involved in TOR and GATOR complexes. Brain somatic activating mutations in *MTOR* have been described in patients with epilepsy caused by focal cortical dysplasia (FCD) type II (Lim and Lee, 2016; Møller et al., 2016). The variants described are located along all exons of *MTOR*, although certain hotspots have been identified (Figure 4J). Recently, the phenotypic spectrum associated with germline variants in *MTOR* was extended to include milder phenotypes than previously reported (Møller et al., 2016). Møller et al. found 5 *de novo* germline mutations in *MTOR* in 6 individuals with variable epilepsy phenotypes (ranging from focal to generalized) and brain malformations (ranging from no malformation to macrocephaly). Moreover, they describe a variant found in a mother-daughter pair with nocturnal epilepsy of the frontal lobe. *MTOR* is therefore also a candidate gene for epilepsy without cortical malformation. Our analyses revealed that this gene was the second best conserved: its low z-score implies a high sensitivity to missense variants (Figure 1). Nonetheless, the CONDEL scores for the variants detected in our cohort do not indicate a high level of deleteriousness compared with controls (Figure 2). Two patients in our cohort carried *MTOR* variants (Table 1B). In the first patient (P918), we detected 2 biallelic missense variants in *MTOR*. The patient's mother, maternal uncle, and brother presented FS. The probability of finding 2 missense rare variants in this gene is 3.98e^−4^. To our knowledge, there are no reports of epilepsy-associated biallelic mutations in this gene. The second patient (P1084) carried the missense K42N variant, which affects a highly conserved nucleotide. A familial study revealed a paternal family history of seizures, and indicated that the variant was inherited from the patient's father. However, we could not demonstrate perfect cosegregation of the variant with the seizure phenotype, indicating variable penetrance. Further genomic and functional studies will be required to corroborate a relationship between these variants and the patient's epileptic phenotype.

#### Incomplete Penetrance

Except in cases of patients with a positive family history, variants detected in dominant genes inherited from unaffected progenitors or with a MAF > 0 were not classified as pathogenic or likely pathogenic, in accordance with the guidelines of the American College of Medical Genetics and Genomics (ACMG). These variants (listed in Table 1B) are thus pending classification. A role in the patient's phenotype cannot be ruled out since incomplete penetrance is not unusual in autosomal dominant epileptic disorders, as described for example in patients with *PRRT2* frameshift mutations (Cloarec et al., 2012; Lee et al., 2012) and mutations in *SCN1A* (Gennaro et al., 2003; Fukuma et al., 2004; Kimura et al., 2005; Mancardi et al., 2006; Depienne et al., 2009), *SCN8A, KCNT1* (Møller et al., 2015), *SLC12A5*, or *DEPDC5* (Baulac, 2016). Potential explanations for incomplete penetrance include parental mosaicism, oligogenic inheritance, and the Knudson 2-hit mechanism (discussed below).

##### The Knudson 2-hit mechanism

Somatic mutational events in the brain are frequent (Hoang et al., 2016). An individual can inherit one germinal variant from one progenitor and subsequent appearance during development of a second variant in the same or another gene can have an additive effect, giving rise to a clinical phenotype. In FCD, reports of brain somatic mutations in genes involved in the mTORC1 pathway, especially activating somatic *MTOR* variants, are increasingly common (Poduri et al., 2013; Lim et al., 2015; Nakashima et al., 2015; Mirzaa et al., 2016; Møller et al., 2016; Marsan and Baulac, 2018). The presence of loss-of-function mutations in *DEPDC5* is the most common cause of familial focal epilepsies. However, only a subset of patients among families with *DEPDC5* mutations develop FCD (Baulac, 2016); other family members appear to present non-lesional epilepsy. Ribierre et al. (2018) demonstrated that a biallelic 2-hit (brain somatic and germline) mutational mechanism in *DEPDC5* causes focal epilepsy with FCD.

##### Digenic inheritance or modifier genes

Mutations in different ion-channel genes can exacerbate or counteract epileptic phenotypes (Glasscock et al., 2007; Hawkins et al., 2011; Klassen et al., 2011; Calhoun et al., 2017; Hasan et al., 2017). These reports suggest that the co-occurrence of 2 mutations in distinct genes that are independently innocuous can give rise to a clinical phenotype and help explain the incomplete penetrance described for many epilepsy-related genes. Functional studies and/or further reports of similar cases will obviously be required to confirm this hypothesis.

Our cohort included several potential examples of digenic inheritance. For example, 3 patients in our cohort carried variant R1928G in *SCN1A* (frequency, 0.0026 in 1000G). In 2 cases (P13 with EIEE and P396 with Dravet syndrome and severe ID) this variant co-existed with a rare missense variant in *CLCN2* (R68H and S123F, respectively). Neither of these *CLCN2* variants are recorded in PVDB or in 1344 Ib-chr, and the first was confirmed to be in trans with the R1928G variant. The probability of finding 2 rare missense variants simultaneously in both genes is 3.99e^−4^. The *CLCN2* encodes the ClC-2 chloride channel. Underscoring the importance of this channel in the brain, biallelic mutations in this gene are associated with leukoencephalopathy with ataxia (MIM_615651). Although there is some evidence linking *CLCN2* mutations to susceptibility to epilepsy, these data remain controversial, and these cases could alternatively be explained by undetected digenic inheritance (Sander et al., 2000; Haug et al., 2003; Kleefuss-Lie et al., 2009; Saint-Martin et al., 2009). In the third case (patient P141), the *SCN1A* variant was present in cis with a second *SCN1A* variant and in trans with a *KCNT1* variant (Prob = 1.99e^−3^). This patient presented neonatal refractory epilepsy (Otahara syndrome), central coordination disturbance, peculiar phenotype, hepatomegaly, and ventriculomegaly.

Another example was the patient P1113, a male with seizures and GDD, carrying a missense *KCNT1* variant, with a frequency in public variant databases that was incompatible with dominant inheritance with full penetrance, accompanied by 2 other ion channel variants in *HCN1* and *CACNA1H*. In this case further functional studies will be required to determine the variant's role in the patient's phenotype (Prob ~ 0).

P676, in addition to carrying a variant in *GABRB1*, carried another variant in *GABBR2* (Table 1B), a gene also implicated in EIEE (EuroEPINOMICS-RES Consortium, 2014). Unfortunately, this patient lives in an institution and their parents could not be contacted to perform a familial study.

Digenic inheritance was clearer in the case of 2 Portuguese siblings, P501 and P968, who presented very different forms of epilepsy. One had a clear molecular diagnosis for a recessive gene (*SLC12A5*) and carried a maternally inherited *SCN1A* variant. The patient's sister, who had a milder phenotype consistent with absence epilepsy that was well-controlled with valproate, carried a combination of inherited heterozygous variants in genes encoding ion channels: *SCN1A* (maternal), *CACNA1A* (paternal), and *SLC12A5*. In individuals of French-Canadian origin, the *SLC12A5* missense variants R952H and R1049C in heterozygosity have been shown to predispose carriers to generalized epilepsy (Kahle et al., 2014; Puskarjov et al., 2014). In those studies, *in vitro* functional expression studies revealed that these variants impair *SLC12A5* function. Moreover, in several cases the variants were inherited from an unaffected parent, consistent with incomplete penetrance. Further evidence from functional studies in mouse models will be required to confirm cases of proposed digenic inheritance.

### Limitations of the Study

A limitation of the targeted resequencing approach is that, unlike WES, it cannot detect novel epilepsy-associated genes. Furthermore, unlike WGS it does not allow detection of mutations in intronic sequences or non-coding RNAs with transcriptional regulation roles which together correspond to 80% of human genome according to ENCODE Project Consortium 2012 (The ENCODE Project Consortium, 2012). Moreover, our approach can detect neither balanced structural variants nor CNVs outside coding regions, both of which can change the regulatory landscape of developmental genes and alter the delicate balance between transcriptional enhancers, silencers, and insulators (Conrad et al., 2010; Spielmann and Klopocki, 2013). Epimutations, which may account for disease in ~20% of neurodevelopmental disorders and congenital anomalies (Barbosa et al., 2018) are also undetectable with this technology. A final limitation of our approach is that mutations in somatic mosaicism may also go undetected in blood samples and it has been shown that somatic mutational events in brain tissue are frequent (Hoang et al., 2016) and a high frequency of mosaic pathogenic variants in epilepsy-associated genes has been demonstrated (Stosser et al., 2018).

## Conclusions

- Well-optimized targeted exome sequencing of a large high number of genes with high mean coverage can be highly beneficial to clinical specialists and to pediatric epileptic patients and their families. By using this approach in our cohort, we were able to establish a genetic diagnosis in ~42% of cases. This in turn alleviates parental anxiety and guilt, helps identify at-risk family members, and can facilitates reproductive decision-making. Furthermore, it provides parents with access to a community with shared experiences, limits the need for counterproductive tests and treatments, and enables selection of the most appropriate antiepileptic therapy.
- The length of the mean diagnostic delay in our cohort (~6 years, measured from the time of onset of clinical signs until molecular diagnosis) underscores the importance of implementing tests of this kind as first-tier diagnostic tools for epilepsy patients.
- To take into account the varying mutational sensitivities of epilepsy-related genes, their specific mutational architecture, the *in-silico* prediction of missense variants (GERP and CONDEL) and filtering variants using a large, well-characterized database of the patient's population is essential to ensure optimal prioritization of the variants detected.
- CNVs account for a considerable percentage of the mutational load in epilepsies (as much as nonsense and frameshift variants). If the mean coverage achieved using our panel had been lower (as is the case with many diagnostic panels), this specific source of mutations would have gone unnoticed.

## Data Availability Statement

The data analyzed for this study can be found in the BioProject repository, accesion number PRJNA551134: https://www.ncbi.nlm.nih.gov/bioproject/PRJNA551134.

## Ethics Statement

This study was approved by the Ethics Committee of the Hospital Santiago de Compostela (Spain). A total of 246 patients were recruited over a 5-year period from different neurologic units in Spain and Portugal, applying the following inclusion criteria: patients with any condition in which seizures are either the sole clinical expression or are part of more severe neurodevelopmental disorder.

## Author Contributions

AF-M designed and optimized successive versions of NGS based epilepsy panels, interpreted genetic data, generated reports, and write the manuscript. IR performed bioinformatic analyses, transforming raw data into an annotated table of prioritized variants, conducted statistical analyses of z-score and GERP and CONDEL scores, contributed to the writing of successive versions of the manuscript, compiled references, and managed variant annotation and optimized figures. The rest of the authors contributed with the clinical care of patients, blood sample collection from patients and their families, collected clinical and family history data, and collaborated in the edition of successive versions of the manuscript.

### Conflict of Interest

The authors declare that the research was conducted in the absence of any commercial or financial relationships that could be construed as a potential conflict of interest.

## Supplementary Material

Supplementary Table 1 Clinical features of patient listed in Table 1.
