# Supplementary material for: Rare Variants in 48 Genes Account for 42% of Cases of Epilepsy With or Without Neurodevelopmental Delay in 246 Pediatric Patients
Source: Front Neurosci. 2019 Nov 8;13:1135. doi: 10.3389/fnins.2019.01135 (PMC6856296; doi:10.3389/fnins.2019.01135)
Supplement: Supplementary Table 1 — Clinical features of patient listed in Table 1. [file Table_1.DOCX]

| PATIENT | SEX | GENE(S) | DX | ONSET | CLINICAL FEATURES |
| --- | --- | --- | --- | --- | --- |
| **13** | M | SCN1A  CLCN2 | 2 m | 13 d | EIEE. |
| **37** | F | PRRT2 | 6 m | Neo | Seizures and a positive maternal family history. |
| **38** | M | PCCA | 9 y | 8 m | Refractory EIEE with features of ASD, severe ID, microcephaly, and dyskinetic cerebral palsy. Initially the patient presented metabolic acidosis, leading to suspicion of a mitochondrial disorder. Multiple carboxylase deficiency had been suspected but never confirmed. |
| **41** | M | KCNT1 | 7 y | 8 m | Malignant migrating epilepsy with onset at 8 months, and severe secondary EIEE, which led physicians to suspect a mitochondrial disorder. |
| **47** | F | CDKL5 | 7 y | 15 m | Focal aware seizures with motor onset of epileptic spasms with evolution to EIEE. ID and ASD. |
| **52** | F | SCN1A | 1 y | 3 w | EIEE. |
| **67** | M | SLC13A5 | 7 y | 4 h | Otahara Syndrome (onset, 4 hours after birth) that evolved to treatment-refractory West Syndrome and severe psychomotor development retardation. |
| **71** | F | SCN1A | ? | Neo | Severe refractory EIEE. |
| **76** | M | SYNGAP1 | 8 y | 10 m | Refractory epilepsy (Lennoux-Gastaut syndrome), DD, PMD, and severe ID. |
| **84** | M | KCNQ3 | 5 m | 2 d | Benign neonatal seizures and positive paternal family history of seizures and cosegregation. |
| **109** | M | KCNB1 | 4 y | <1 year | Refractory EIEE, acquired microcephaly, axial hypotonia, PMD, GDD, poor manipulation skills, hand-mouth stereotypies, and absence of language. |
| **131** | F | CDKL5 | 9 m | NA | EIEE, severe PMD, and divergent strabismus. |
| **141** | F | SCN1A/KCNT1 | 7 m | Neo | Refractory epilepsy (Otahara syndrome), alteration of central coordination, peculiar phenotype, hepatomegaly, and ventriculomegaly. |
| **156** | F | CDKL5 | 13 y | 5 m | Epileptic spams with evolution to EIEE and Rett-like regression. |
| **164** | M | SLC9A6 | 2 y | 7 m | EIEE, acquired microcephaly, global PMD, absence of proactive language, hand-mouth stereotypies, and axial hypotonia. |
| **186** | F | ARHGEF9 | 5 y | 15 m | EIEE, PMD, and GDD. |
| **201** | F | DNM1 | 1 y | NA | PMD and seizures. |
| **202** | F | CDKL5 | 5 y | NA | EIEE and PMD. |
| **207** | F | PRRT2 | NA | 5 m | Benign form of epilepsy and positive family history. |
| **218** | F | GRIN2A | 15 y | 6 y | Refractive multifocal epilepsy, moderate psychomotor delay, language problems, ID, and fine motor skill deficits. |
| **248** | F | SCN9A | 4 y | 2.5 y | Secondary partial epilepsy unresponsive to treatment, PMD with Rettoid features. Left parieto-occipital  type II dysplasia, 2 interventions. |
| **268** | M | KCNT1 | 27 d | Neo | Hard-to-control seizures and a severely abnormal background electroencephalogram pattern. |
| **289** | M | CSNK2B | 12 y | 3 m | Seizures, GDD, and mild ID. |
| **310** | M | HNRNPU | 15 y | 6 m | FS, which progressed to EIEE (Lennox-Gastaut syndrome) and severe PMD. No evidence of renal or cardiological pathology. |
| **314** | F | SCN2A | 8 y | 17 m | EIEE, altered sleeping pattern, GDD, agitation, stereotypies, and lower limb spasticity. |
| **317** | F | GNAO1 | 3 y | 1 m | Severe EIEE with dystonia, complex I deficiency in fibroblasts, and mitochondrial proliferation in muscle tissue. |
| **379** | F | SCN2A | 15 d | NA | EIEE associated with severe PMD. |
| **385** | M | MECP2 | 6 y | 1 y | EIEE (Lennox-Gastaut syndrome) and severe PMD. |
| **396** | M | SCN1A | 29 y | <1 y | EIEE (Dravet syndrome), severe ID. |
| **406** | F | SCN1A | 9 y | NA | Severe EIEE. |
| **420** | F | FOXG1 | 11 y | Neo | Seizures, severe PMD, microcephaly, and generalized hypotonia of axial predominance. |
| **421** | M | TBC1D24 | 4 m | Neo | Onychodystrophy, deafness, and seizures. |
| **427** | M | FOXG1 | 7 y | <1 y | Microcephaly, EIEE, severe ID, and neuronal migration defect. |
| **472** | M | CDKL5 | 21 m | 4 m | EIEE, severe PMD, spastic-dystonic infantile cerebral palsy, movement disorder, and muscle biopsy with inconclusive findings suggestive of mitochondrial disorder. |
| **473** | F | CDKL5 | 5 m | 15 d | Treatment-resistant focal motor onset behaviours with impaired awareness, PMD. |
| **474** | F | MECP2 | 24 y | 2 y | GDD, ID, and generalized unresponsive epileptic syndrome. Periventricular leukomalacia. |
| **475** | F | KCNQ2 | 2 m | NA | EIEE unresponsive to treatment. |
| **481** | M | DNM1 | 4 y | NA | EIEE, progressive microcephaly, GDD, and hypotonia. |
| **485** | F | KCNQ2 | 1 y | NA | FS evolving to partial complex crises refractory to treatment, PMD and ADHD, mesial sclerosis. |
| **501** | M | SLC12A5 /SCN1A | 12 y | Neo | Malignant migrating seizures, microcephaly with global atrophy, hypomyelination, and a thin corpus callosum*.* |
| **519** | M | RBFOX1 | 6 m | 4 m | EIEE. |
| **523** | F | STXBP1  SIK1 | 32 y | NA | "Treatment-refractory epilepsy"? |
| **528** | F | SCN1A | 24 y | NA | PMD and epilepsy unresponsive to treatment. |
| **530** | M | GPHN | 6 y | NA | Severe GDD, with stereotypies, autism, and seizures. This patient’s mother was unaffected, but his maternal uncle shared clinical features with the index case. |
| **545** | F | ARX | 7 y | Neo | Intractable epilepsy, ID, ADHD, immature visuomotor function, altered social skills, and motor clumsiness. |
| **548** | F | SCN1A | 4 y | 21 y | Generalized epilepsy with FS ++. Brother with simple FS. |
| **561** | M | RFT1 | 19 y | <1 y | EIEE with PMD, severe ID, microcephaly, stereotyped hand movements, spastic tetraparesis, and a complete absence of language. Other clinical signs included a syndromic appearance with an anteverted nose and an everted lower lip. Normal serum transferrin pattern. |
| **571** | M | KCNQ2 | 4 y | Neo | Severe EIEE and generalized hypotonia. Mimics mitochondrial disorder. |
| **572** | M | MECP2 | 6 y | NA | Progressive hypotonia, abnormal eye movements, PMD, and symptomatic epilepsy. Deceased sibling with a similar condition. |
| **597** | F | SCN1A | 12 y | NA | EIEE (Dravet-like) and joint hypermobility. |
| **598** | M | STXBP1 | 7 y | NA | Treatment refractory EIEE and severe PMD. |
| **606** | M | GNAO1 | 14 y | 6 y | Seizures and paroxistic head movements. |
| **629** | M | PRRT2 | 1 y | 6 m | Benign form of epilepsy and positive family history. |
| **650** | M | FOXG1 | ? | 1 y | Myoclonic epilepsy, language delay, and behavioural disorder (aggressiveness). No microcephaly or stereotypes. Presence of neurodevelopmental disorder but no regression. |
| **651** | M | SCN1A | ? | NA | PMD and epilepsy unresponsive to treatment. |
| **652** | M | SCN1A | 11 m | 5 y | Status epilepticus (Dravet syndrome). |
| **658** | M | HCN1 | 3 m | 2 d | Refractory EIEE that evolved to status epilepticus. Patient died at 15 months of age. |
| **660** | M | SCN8A | 9 m | Neo | Hypotonia, West syndrome, and GDD. |
| **676** | M | GABRB1 | NA | NA | NA |
| **684** | F | GNAO1 | 3 y | 5 m | Severe EIEE, dystonia, DD, hyperlactacidaemia, and psychomotor regression. |
| **685** | M | RHOBTB2 | 6 y | 5 m | Severe refractory seizures, severe PMD, and acquired microcephaly. |
| **703** | M | SCN2A | 3 y | 3 y | Partial complex myoclonic epilepsy, without neurological involvement. |
| **704** | F | KCNQ2 | 2 y | NA | EIEE, cortical atrophy, and progressive leukoencephalopathy. Mimics mitochondrial disorder. |
| **716** | F | SCN1A | 2 y | NA | Severe EIEE. |
| **738** | F | FOXG1 | 18 y | NA | Epilepsy + dyskinesia. |
| **740** | M | SYN1 | 11 y | 5 y | Uncontrolled epilepsy (complex partial crises), ID, and PMD. Born to a mother with ID. |
| **744** | F | KCNQ2 | 14 y | 11 y | Generalized epilepsy (myoclonic and tonic-clonic seizures). |
| **754** | M | GABRB1 | 6 y | 7 m | Treatment-refractory EIEE, microcephaly, and frontal brain atrophy. Paternal grandfather, uncle developed seizures at 3-4 years of age. |
| **759** | F | SCN2A | ? | 5 d | Uncontrolled epileptic seizures evolving to status epilepticus. Died at 1 month of age. |
| **781** | M | SCN2A | 8 m | Neo | Seizures, positive maternal family history of FS with autosomal dominant pattern. |
| **782** | M | SRGAP2 | 16 y | 5 m | Clonic convulsions of the extremities with onset 2 days after birth, evolving to brief tonic spasms. Hypsarrhythmia on electroencephalogram was detected at 5 months of age, indicating a transition to West syndrome. |
| **801** | F | SCN1A | 4 y | NA | EIEE (West and Lennox syndromes). |
| **819** | M | GPHN | 2 y | 11 m | FS, language impairment, and benign evolution during follow up. Mother was asymptomatic. |
| **829** | M | SCN1A | 7 y | NA | FS. |
| **832** | M | SCN1A | 1 y | <1 y | Afebrile and febrile generalized tonic-clonic convulsive seizures with low temperature. |
| **857** | F | ARHGEF15 | 9 y | NA | Epilepsy with apraxia. |
| **859** | F | SLC35A2 | 9 y | 1,5 m | EIEE (Otahara syndrome), GDD, delayed myelination, arachnoid cyst, agenesis/hypoplasia of the right olfactory bulb, dysmorphic facial features, contractures of fingers, and overlapping toes. |
| **860** | F | SCN1A | 9 y | <1 y | Refractory epilepsy. |
| **868** | F | KCNA2 | 17 y | 11 m | Treatment-refractory seizures, mild language retardation, strabismus, mild ataxia, joint hyperlaxity, and generalized hyperreflexia. |
| **872** | M | FOXG1 | 11 y | 6 m | Stereotypic activities and epilepsy that was controlled with medical treatment. Profound PMD, progressive microcephaly, and spastic tetraparesis. |
| **875** | M | KCNQ2 | 1 m | Neo | Difficult to control seizures, but the crisis subsided. Father experienced convulsions within the first 6 months of life. |
| **884** | F | ARHGEF9 | 16 y | NA | EIEE. |
| **887** | M | SCN1A | 5 y | NA | EIEE. |
| **901** | M | CLCN4 | 18 y | 1 y | Idiopathic epilepsy and mild ID. Patient’s brother and maternal uncle had a similar phenotype, indicating cosegregation. |
| **904** | M | GABRG2 | 14 y | 5 m | FS evolving to complex partial seizures, absences, generalized tonic-clonic seizures responsive to levetiracetam, ADHD, and mild ID. The patient’s father and aunt had FS up to 4–5 years of age. |
| **918** | M | MTOR | 1 y | 7 m | Seizures with a right temporal focus on cerebral magnetic resonance imaging and electroencephalogram. Mother, maternal uncle, and brother with convulsive FS. |
| **937** | M | SCN1A | 6 m | 1 m | Polymorphic seizures, DD. |
| **938** | F | GABRB1 | 10 y | 2 y | Seizures (onset, 2 years of age), GDD, and ID. Patient had the same dysmorphic facial features as her mother. |
| **951** | M | DEPDC5 | 13 y | 3 m | West syndrome and severe GDD. Sister with focal epilepsy. |
| **954** | M | PROSC | 10 m | <1 y | Frequent seizures and associated acute illness, with onset in the first year of life. The patient’s response to pyridoxine could not be evaluated as he had been administered this drug since beginning treatment. |
| **957** | M | KCNT1 | 19 y | NA | Refractory EIEE secondary to focal epilepsy and ID. |
| **965** | F | MECP2 | 13 y | NA | EIEE with prior ID. |
| **968** |  | SCN1A/ SLC12A5/ SCN10A | 13 y | NA | Milder phenotype consistent with absence epilepsy, well controlled with valproate. Sibling of patient 501. |
| **975** | M | PRRT2 | NA | NA | Benign form of epilepsy and positive family history. |
| **980** | M | SCN1A | 20 y | 9 m | FS that evolved to generalized complex crises. Manual and oroalimentary automatisms. Mother, maternal aunt, and cousin with FS. Sister with FS with onset at 12 years of age. Paternal cousin with FS with onset at 2 years. |
| **981** | M | HCN1/ GRIN1 | 3 y | 3 y | Seizures resembling occipital epilepsy and behavioural alterations. Family history of epilepsy (paternal grandfather). |
| **996** | M | NPRL2 | 4 y | NA | Frontal seizures. Father with focal epilepsy. |
| **1008** | M | KCNT1 | 11 y | 2.5 y | Treatment-refractory, apparently frontal nocturnal seizures. Learning difficulties. |
| **1029** | M | GRIA3 | 21 y | NA | Epileptic seizures (atypical West syndrome), ID, GDD, ASD, and behavioural disorder. |
| **1030** | F | FGF12 | 13 y | <1 y | Severe EIEE, low CSF folate levels, and severe osteoporosis, leading to initial suspicion of a Glut1 defect. |
| **1038** | M | SCN8A | 11 m | NA | Refractory focal epilepsy and positive family history. |
| **1049** | M | CNTNAP2 | 5 y | NA | Acquired microcephaly, ASD, epilepsy, and growth retardation. |
| **1058** | M | IL1RAPL1 | 7 y | Neo | Status epilepticus and GDD. |
| **1068** | M | KCNC1 | NA | 3 y | Generalized epilepsy-like absence seizures that were partially responsive to valproate. Patient has had no clinical crises since November 2015. |
| **1084** | M | MTOR | 12 d | Neo | Seizures and paternal family history of seizures. |
| **1085** | M | KCNQ2 | 1 m | Neo | Seizures refractory to treatment, poor general condition, distress, hyperammonaemia, severe metabolic acidosis, suspected mitochondrial disorder. |
| **1099** | M | HECW2 | 3 y | NA | EIEE and GDD. |
| **1112** | M | SCN1A | 2 y | 16 m | Seizures responsive to treatment. Father and cousin with a history of FS in childhood. |
| **1113** | M | KCNT1 | 15 y | NA | GDD, seizures. |
| **1119** | F | SYN1 | 12 y | NA | Treatment-responsive generalized epilepsy with absences, language delay, ID, and movement disorder. |
| **1126** | M | SLC6A1 | 9 y | NA | GDD with epilepsy, language impairment, ADHD, and peculiar phenotype. |
| **1127** | F | CDKL5 | 9 m | 3 m | Tonic and clonic seizures and PMD. |
| **1129** | F | PRRT2 | 1 y | <1 y | Benign form of epilepsy and positive family history. |
| **1144** | M | TSC2 | 2 m | Neo | Tonic spasms, altered EEG, and normal MRI. Response to vigabatrin was typical of patients with TSC. |
| **1156** | F | SLC6A1 | 7 y | NA | GDD with epilepsy, language impairment, ADHD, and peculiar phenotype. |
| **1173** | M | SCN10A | 10 y | NA | Refractory epilepsy and ASD. |
| **1177** | M | CLCN4 | 14 y | NA | Dravet syndrome-like features that were controlled with topiramate, as well as ID and difficulties communicating and socializing. |
| **1197** | M | DHDDS | 17 y | 5 y | PMD, seizures, and tonic rigidity. |
| **1198** | M | SCN1A | 15 y | 5 m | EIEE (Lenox-Gastaut syndrome), ID, and spasticity. |
| **1208** | F | GNAO1 | NA… | NA | NA |
| **1213** | M | NPRL3 | 7 y | NA | Partial seizures and language impairment. Family history of seizures (father and paternal aunt). |
| **1214** | M | STXBP1 | 9 y | 1 m | Refractory epilepsy, severe PMD, absent speech, and limited social skills. |
| **1233** | F | STXBP1 | 9 m | 80 h | Focal seizures and PMD. |
| **1238** | M | WWOX | 2 m | Neo | Horizontal nystagmus and left colpocephaly, hard-to-control generalized seizures, thumb-in-fist posture, clinodactyly, micrognathia, acute respiratory stridor, difficulties swallowing, and hyperflexioned posture. |

Abbreviations: ADHD, attention deficit hyperactivity disorder; ASD, autism spectrum disorder; d, days; Dx: age at molecular diagnosis; EIEE, early infantile epileptic encephalopathy; FS, febrile seizures; GDD, global developmental delay; ID, intellectual disability; m, months; MRI, magnetic resonance imaging; Neo, neonatal; Onset, age at onset; PMD, psychomotor delay; TSC, tuberous sclerosis; w, weeks; y, years.
